# Cell composition inference and identification of layer-specific spatial transcriptional profiles with POLARIS

**DOI:** 10.1126/sciadv.add9818

**Published:** 2023-03-01

**Authors:** Jiawen Chen, Tianyou Luo, Minzhi Jiang, Jiandong Liu, Gaorav P. Gupta, Yun Li

**Affiliations:** ^1^ Department of Biostatistics, University of North Carolina at Chapel Hill, Chapel Hill, NC, USA.; ^2^ Department of Applied Physical Sciences, University of North Carolina at Chapel Hill, Chapel Hill, NC, USA.; ^3^ Department of Pathology and Laboratory Medicine, University of North Carolina at Chapel Hill, Chapel Hill, NC, USA.; ^4^ Department of Radiation Oncology, University of North Carolina at Chapel Hill, Chapel Hill, NC, USA.; ^5^ Lineberger Comprehensive Cancer Center, University of North Carolina at Chapel Hill, Chapel Hill, NC, USA.; ^6^ Department of Genetics, University of North Carolina at Chapel Hill, Chapel Hill, NC, USA.; ^7^ Department of Computer Science, University of North Carolina at Chapel Hill, Chapel Hill, NC, USA.

## Abstract

Spatial transcriptomics (ST) technology, providing spatially resolved transcriptional profiles, facilitates advanced understanding of key biological processes related to health and disease. Sequencing-based ST technologies provide whole-transcriptome profiles but are limited by the non–single cell–level resolution. Lack of knowledge in the number of cells or cell type composition at each spot can lead to invalid downstream analysis, which is a critical issue recognized in ST data analysis. Methods developed, however, tend to underuse histological images, which conceptually provide important and complementary information including anatomical structure and distribution of cells. To fill in the gaps, we present POLARIS, a versatile ST analysis method that can perform cell type deconvolution, identify anatomical or functional layer-wise differentially expressed (LDE) genes, and enable cell composition inference from histology images. Applied to four tissues, POLARIS demonstrates high deconvolution accuracy, accurately predicts cell composition solely from images, and identifies LDE genes that are biologically relevant and meaningful.

## Teaser

POLARIS can perform cell type deconvolution, identify LDE genes and enable cell composition inference from histology images.

## INTRODUCTION

Molecular analysis of mRNA patterns in histological tissue sections is a key component of biomedical research and diagnostics. The development of novel spatial transcriptomic (ST) technologies has advanced dramatically over the past few years. There are two main categories of ST technologies: imaging-based or sequencing-based. Technologies based on imaging directly image individual RNA molecules within single cells (*1*, *2*). Sequencing-based techniques first label spatial spots on histological tissue sections with unique barcodes to indicate their two-dimensional spatial positions, and use RNA sequencing (RNA-seq) to provide gene expression quantifications for each spot along with the spatial coordinates (*3*, *4*). Commonly used methods include MERFISH (*1*), seqFISH+ (*5*) in the former category, and 10X Genomics’ Visium platform (*3*) in the latter category. More information can be found in recent review papers (*6*–*8*). Some of the sequencing-based techniques (exemplary platforms include Spatial Transcriptomics and Visium) also provide a co-registered hematoxylin and eosin (H&E)–stained histology image for the analyzed sample. Empowered by these technologies, we can obtain gene expression profiling with retained spatial information and histological images, which enable researchers and clinicians to gain an improved level of insight into complex tissue samples.

In parallel to these technological developments, computational methods to analyze spatial data derived from tissue samples have substantially advanced. For instance, focusing on histology images, multiple machine learning and deep learning methods have been developed to maximally extract information from these images (*9*–*12*). In the presence of pathological annotations, histology images can be used for various purposes including cell segmentation (*13*, *14*), tissue type registration (*15*), mutation rate inference (*16*, *17*), and gene expression prediction (*12*). Most of these tasks, however, require pathologists to fully annotate each cell in the histological image, entailing substantial manual time and human resources. This pathologist annotation is currently unavailable for the vast majority of publicly available ST data, thus making using traditional cell detection methods to perform ST deconvolution inaccessible. In the field of ST, histology imaging has primarily been used to predict gene expression and perform tissue registration, where the image data are usually subject to a pretrained model to extract image features (*9*, *10*, *12*). Several popular pretrained models, such as convolutional neural networks, stacked sparse autoencoders, and masked autoencoders (MAEs), have been used as a first step to reduce image dimensions and demonstrate advantages in many applications (*9*, *10*, *12*, *13*, *15*). However, cell composition inference has not benefited from these models yet. In recent literature, histology images have been used to improve deconvolution accuracy (*18*, *19*), but methods that can predict cell composition solely from histology images are currently unavailable.

**Fig. 1.**
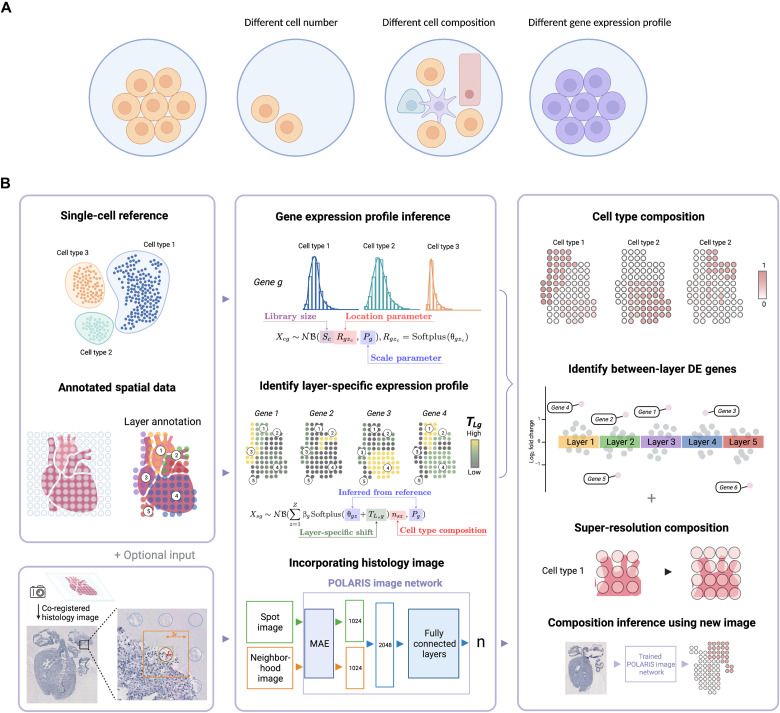
POLARIS overview. (**A**) Three reasons that can explain gene expression variation across spots. Each circle represents a spatial spot. Compared to the leftmost spot, the three spots on the right differ primarily in cell number, cell composition, and gene expression profile. (**B**) POLARIS workflow. POLARIS takes as input single-cell reference and annotated spatial data, infers cell type–specific gene expression profiles, and identifies layer-specific expression profiles. When a co-registered histology image (as an optional input) is provided, POLARIS will additionally train an image network. The output of POLARIS includes inferred spot-level cell type composition, identified LDE genes, and a pretrained POLARIS image network that can be applied to independent images. This figure is created via BioRender.

Besides the histology image, ST data allow for the extraction and revelation of tissue structure through coordinated gene expression. Researchers have developed methods such as SPARK (*20*) and SpatialDE (*21*) for identifying genes whose expression varies within a tissue slice, known as spatially differentially expressed (SDE) genes. Gene expression changes spatially across spots within a tissue slice, often reflecting some underlying structured heterogeneity such as anatomical layers, clusters of similar spots, and/or spatial domains. This structured heterogeneity motivates the development of ST clustering methods including BayesSpace to identify layers/clusters within each ST slice (*11*, *22*, *23*). As aforementioned, the identified layers often correspond to different functions or morphological changes in the tissue (*22*, *24*, *25*). The across-spot variation in expression can be largely attributed to three factors: variation in cell number, variation in cell composition, and true spatially driven variation in gene expression profile (Fig. 1A).

When the expression variation is truly driven by difference in spatial coordinates (in contrast to difference in cell number or composition), we can consider having sub-cell types located in different spatial regions (Fig. 1A). However, when SDE genes are detected by the aforementioned methods, the identified spatial difference is a result of the interplay of all three factors, and it is difficult to distinguish genes that are truly SDE from those that merely appear so due to differential cell number or composition across spatial spots. We would be able to differentiate among the driving factors if we had single-cell resolution data with the entire transcriptome or at least a large number of genes measured. However, in practice, we normally do not have this luxury: We have either data from imaging-based technologies that are single-cell resolution but measure only a small number of genes or data from sequencing-based methods that provide transcriptome-wide measurement but are limited in resolution. Sequencing-based ST methods have spatial spots of 2 to 100 μm in diameter, implying that each spot can easily contain tens of cells of different cell types. Lacking ideal (i.e., single-cell resolution with many genes measured) data motivates the development of computational methods to infer variation in gene expression profile across layers while simultaneously estimating and adjusting for the estimated cell number and composition.

As a matter of fact, lack of knowledge in the number of cells at each spot or the cell type composition itself has been recognized as a critical issue in ST data analysis because failure to adjust for this accurately can lead to invalid downstream analyses. To address this problem, a number of ST deconvolution methods have been developed (*18*, *26*–*30*). However, most ST deconvolution methods assume that the gene expression profile for the same cell type is invariant across the entire tissue sample, which is a strong assumption whose violation will result in inaccurate cell composition inference. Methods such as DestVI that assume a continuous or smoothly changing gene expression profile across the tissue, however, have exhibited inconsistent performance across tissue types (*6*, *31*). Therefore, how to model layer-specific gene expression variation and use histological images to infer cell composition is a problem that remains unsolved.

Here, we present POLARIS (Probabilistic-based cell cOmposition inference with LAyer infoRmatIon Strategy) to perform cell type deconvolution and infer layer-wise differentially expressed (LDE) genes (Fig. 1B). POLARIS integrates single-cell RNA-seq (scRNA-seq) reference and ST data with annotated layer information. By examining histology images and the coordinated expression profile, one can reasonably infer layers or subregions that correspond to different biological functions (e.g., cancer versus noncancer regions in a tumor biopsy, different layers in a brain cortical sample, and ventricle and atrium areas in heart). By explicitly allowing and modeling layer-specific gene expression patterns, POLARIS not only is capable of identifying cell type composition with high accuracy but also could identify LDE genes while simultaneously correcting for differential cell composition. An additional key characteristic of POLARIS is its flexibility to optionally leverage histology images. To our knowledge, POLARIS is the first ST deconvolution method that can predict cell composition purely from a histological image. This functionality also empowers POLARIS to infer super-resolution cell composition based on images of areas without gene expression measurements (i.e., areas in between spots) as well as to predict cellular composition based purely on an original H&E-stained image. The performance of POLARIS was evaluated on data from multiple tissues including the mouse cortex, developing human heart, and human epidermal growth factor receptor 2–positive (HER2+) breast cancer samples. POLARIS robustly demonstrates high deconvolution accuracy across tissues compared to other state-of-the-art deconvolution methods, accurately predicts cell composition solely from images, and identifies LDE genes that are biologically relevant and meaningful. Our results showcase the advantages of POLARIS in the following three aspects: deconvolution accuracy, LDE gene identification, and prediction with image.

## RESULTS

### POLARIS method overview

POLARIS is a probabilistic-based inference method that assumes that gene expression counts in both scRNA-seq reference data and ST data follow a negative binomial distribution. As a first step, POLARIS maximizes likelihood to infer cell type–specific gene expression profiles from scRNA-seq reference (Fig. 1B). The gene expression profile of each spot in ST data can then be viewed as a weighted sum of the negative binomial distribution derived from the scRNA-seq reference, where the weights are based on spot-level cell composition. As opposed to assuming that cell type–specific gene expression profiles are invariant throughout a whole tissue slice, POLARIS assumes that only spots in similar biological or anatomical layers share the same gene expression profiles by introducing a layer-specific location parameter. Explicitly modeling layers is a unique feature of our POLARIS method. POLARIS accepts any user-specified layer annotations, e.g., derived manually (from pathologist annotation) or computationally [based on either morphological features or gene expression, e.g., using BayesSpace (*22*)]. Note that the layer-specific parameters cannot be inferred from single-cell reference because there is no layer information by the nature of data generation. Using ST data with layer annotations, POLARIS enables layer-specific inference. By introducing the layer-specific shift parameters (Supplementary Materials), we can obtain an updated location parameter for each layer in the ST data, allowing cell type–specific gene expression profiles to vary across layers. By multiplying the updated location parameter with the cell composition parameter as well as the parameter to account for technical/batch effects, we simultaneously model the impact of cell composition and spatial location (as reflected by layers) on cell type–specific gene expression while controlling for potential batch effects. Parameters can be estimated using maximum a posteriori (MAP) estimation (Supplementary Materials). So far, we have focused on inference with gene expression data only. When a co-registered histology image is available, POLARIS first uses MAE (*32*) to extract features from the image tile of each spot and the image tile of its neighborhood. These two extracted features are then combined and used as inputs to build POLARIS’s image network (Fig. 1B). The output of POLARIS’s image network is cell composition for any input image (which can be from a completely independent histological image). The output of POLARIS includes inferred spot-level cell type composition and layer-specific gene expression profiles, as well as a trained POLARIS image network. The layer-specific gene expression profiles enable identification of LDE genes, and the pretrained POLARIS image network allows resolution enhancement and cell composition inference from a new histology image.

### POLARIS attains high deconvolution accuracy

The deconvolution accuracy of POLARIS was assessed both through simulation and in single-cell resolution ST data. Specifically, we simulated cells with gene expression counts from cell type– and layer-specific negative binomial distributions and randomly selected cells to create spot-level gene expression. For single-cell resolution real ST datasets, we clumped cells into spots according to their coordinates to mimic low-resolution spot-level ST data. We used data where we have cell type labels for the single cells such that we have the true cell type mixture in each clumped pseudo-spot. We quantified the performance using root mean squared error (RMSE) where a smaller RMSE corresponds to better performance. We compared POLARIS with five state-of-the-art methods: CARD (*26*), DestVI (*29*), RCTD (*28*), stereoscope (*27*), and SPOTlight (*30*). These methods were selected according to their specific methodological features and/or their high performance in previous benchmarking studies (Supplementary Materials) (*6*, *31*).

**Fig. 2.**
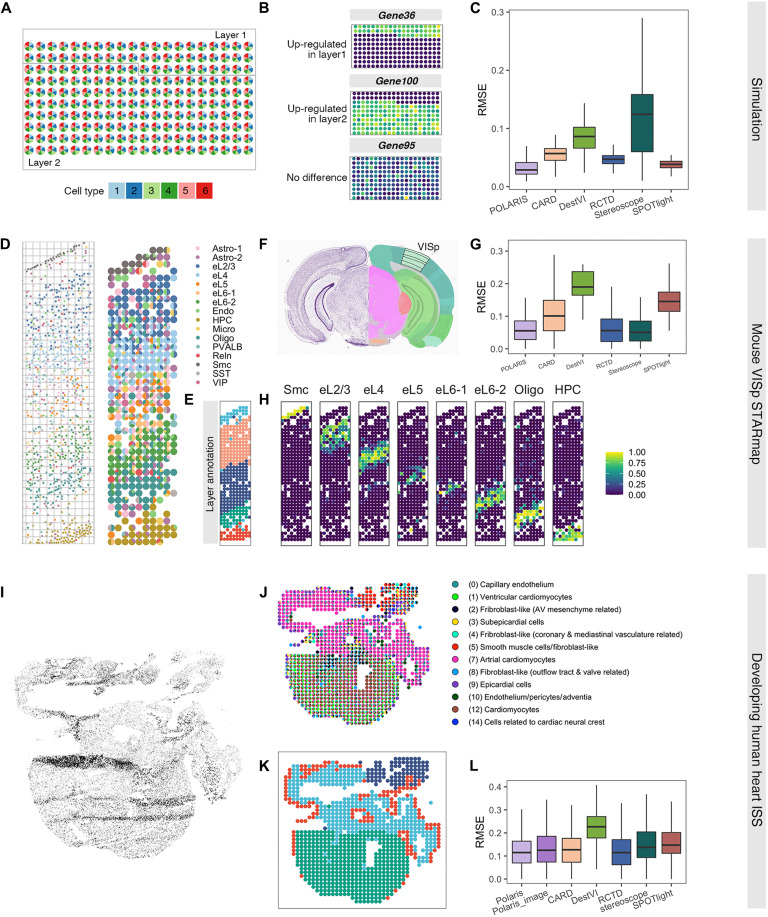
Deconvolution accuracy of POLARIS. Simulation: (**A**) Cell type composition of the simulated ST data. (**B**) Three categories of gene expression pattern: up-regulated in layer 1, up-regulated in layer 2, and no substantial difference across layers. (**C**) RMSE of POLARIS along with other state-of-the-art methods on simulated data. On mouse VISp STARmap data: (**D**) Cell map (left) and clumped pseudo-spots (right) along with their cell compositions visualized by pie charts. (**E**) BayesSpace identified clusters, consistent with layer structure of mouse VISp. (**F**) Nissl staining (left) and anatomical annotations (right) from the Allen Mouse Brain Atlas and Allen Reference Atlas—Mouse Brain. The black lined area indicates the layer structure of the VISp region. (**G**) RMSE of POLARIS along with other state-of-the-art methods on mouse VISp STARmap data. (**H**) POLARIS-inferred spot-level composition of the eight major cell types, along cortex depth. On developing human heart ISS data: (**I**) Processed DAPI-stained histology image of the developing human heart ISS data. (**J**) Clumped pseudo-spots in the ISS data along with their cell compositions visualized by pie charts. (**K**) BayesSpace identified clusters in the ISS data. (**L**) RMSE of POLARIS along with RMSE of other state-of-the-art methods on the heart ISS data.

We began with a simulated scenario where all spots and layers share a similar composition of cells, but with layers differing in terms of their gene expression profiles. Under this scenario, gene expression variations are solely the result of variations in gene expression profiles across layers. Specifically, we first simulated a dataset with two “biological” layers, with cells from six cell types and expression values for 100 genes generated. We first simulated the layer of each cell, and then the gene expression values for the cell were drawn from negative binomial distributions according to its layer and cell type. We then constructed pseudo-spots by randomly selecting 10 to 16 cells from each layer. Specifically, we generated 200 spots with 50 spots in layer 1 and 150 spots in layer 2 (Supplementary Materials). Under this simulation framework, genes could be classified into three categories: up-regulated in layer 1 (e.g., *Gene36*), up-regulated in layer 2 (e.g., *Gene100*), and no substantial difference between layers (e.g., *Gene95*) (Fig. 2, A and B, and fig. S1). The largest log_2_ fold change is seen in *Gene36* and *Gene100*, and most genes display similar or nondifferential expression patterns across layers. Applied to the simulated data, POLARIS outperforms all other methods as manifested by its lowest RMSE (Fig. 2C).

We further assessed POLARIS’s deconvolution performance in a real single-cell resolution ST data from the mouse primary visual cortex (VISp) region, a well-structured region in mouse cortex that has been extensively studied (*2*, *33*). Anatomical structure, major cell types, and layer-specific gene markers provide information about the layered and segmented structure of the mouse VISp (Fig. 2, D to F) (*33*, *34*). We used single-cell resolution ST data from the STARmap platform (*2*), which consists of 1020 genes measured in 973 cells. We divided the cells into 356 pseudo-spots each of 400 × 400 square pixels (Fig. 2D). To perform deconvolution, we used the internal reference (that is, the STARmap single-cell data itself as the reference). In this way, any systematic differences between the reference and the target ST data are eliminated as potential factors that may impair performance. This internal reference evaluation provides a baseline (or upper bound) for measuring the performance of deconvolution methods (*6*). Because layer annotation is required when using POLARIS, we used BayesSpace (*22*) to cluster the constructed pseudo-spots, resulting in five distinct clusters, reflecting the expected layer structure of mouse VISp (Fig. 2E). In this mouse VISp dataset, POLARIS still achieves among the best performance in terms of RMSE (Fig. 2G). Moreover, POLARIS, based on its inferred cellular composition, successfully recovers the layer structure of mouse VISp (from top to bottom: Smc, eL2, eL3, eL4, eL5, eL6-1, eL6-2, Oligo, and HPC; Fig. 2H).

In addition, we tested POLARIS on the developing human heart tissue generated from the in situ sequencing (ISS) platform (Fig. 2I) (*35*). The heart ISS data are also a single-cell resolution ST data, consisting of 24,371 cells and with only 65 genes measured in each cell. We gridded the cells into pseudo-spots, each of dimension 454 × 424 square pixels (Fig. 2J). The main purpose of this assessment is to evaluate POLARIS’s performance with a limited number of genes. Instead of using the internal reference (ISS data itself), we used a scRNA-seq reference obtained from a similar biological sample (*36*). Consequently, we can also evaluate the deconvolution performance when the reference and ST data are not perfectly matched. The heart ISS data provide us with a 4′,6-diamidino-2-phenylindole (DAPI)–stained histology image, allowing us to measure the performance of POLARIS by including the histology image as an additional input (Fig. 2I and Materials and Methods). We clustered the spots into four layers using BayesSpace (Fig. 2K). The BayesSpace inferred layers correspond reasonably well to the anatomy of the heart (red, epicardium; green, ventricles; light blue, atria; dark blue, outflow tract). POLARIS has maintained its best performer position. Specifically, POLARIS achieves the lowest/best mean of MSE (Fig. 2L). Despite the fact that the DAPI staining only contains one color channel, POLARIS with image input is able to effectively infer the type of cell, achieving accuracy close to the best performers.

### Polaris identifies layer-specific gene expression pattern

A major feature of POLARIS is its ability to model layer-specific parameters. The layer/structure of a tissue can be reflected in multiple dimensions, such as morphology, gene expression, and other omics levels. POLARIS focuses on leveraging the rich gene expression information provided by ST data. As detailed above, cell density, cellular composition, and the “real” SDE genes can all contribute to the observed gene expression variation. By incorporating layer-specific parameters into the cell type deconvolution process, POLARIS is able to identify these LDE genes while taking into account differential cell composition. POLARIS quantifies statistical significance for LDE genes using permutation tests, and magnitude of effect using log_2_ fold change in mean gene expression, based on the inferred layer-specific mean parameters (Materials and Methods). Through the elimination of potential confounding effects of cell composition, POLARIS ensures that the LDE genes identified are differentially expressed genes truly due to spatial factors.

**Fig. 3.**
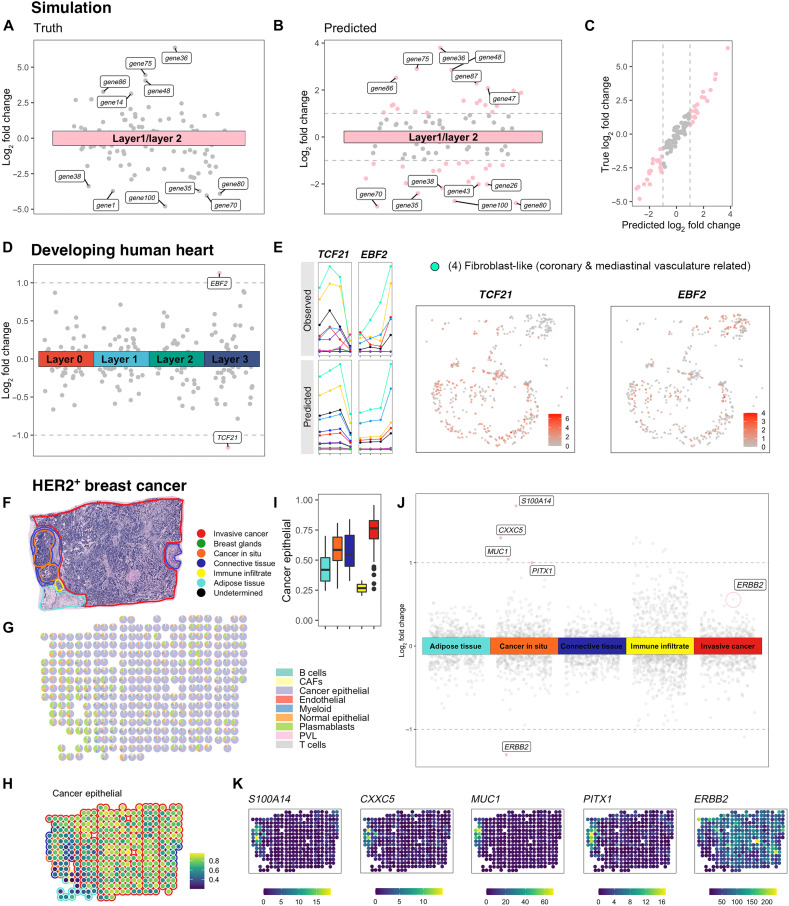
LDE genes identified by POLARIS on three datasets. On simulated data: (**A**) True log_2_ fold change of mean gene expression, layer 1 over layer 2. (**B**) POLARIS-inferred log_2_ fold change of mean gene expression, layer 1 over layer 2. POLARIS-identified LDE genes are colored as pink, otherwise gray. (**C**) POLARIS-inferred log_2_ fold change of gene expression across layers achieves high correlation (Pearson’s correlation = 0.974) with the true log_2_ fold change. On developing human heart data: (**D**) POLARIS-inferred log_2_ fold change of gene expression across layers. POLARIS-identified LDE genes are colored as pink, otherwise gray. (**E**) Left: Observed mean gene expression in scRNA-seq data (top) and POLARIS-inferred gene expression location parameter (bottom) of each cell type across layers. Lines are colored by cell types. *X* axis indicates layer status: From left to right is layer 0, 1, 2, and 3. Right: Gene expression of *TCF21* and *EBF2* in the fibroblast-like (coronary and mediastinal vasculature related) cells. On HER2+ breast cancer data: (**F**) Pathologist annotation on slide A1. (**G**) POLARIS-inferred cell composition. (**H**) POLARIS-inferred cancer epithelial cell proportion. (**I**) Distribution of POLARIS-inferred cancer epithelial cell proportions in each layer [color scheme is the same as in (E)]. (**J**) POLARIS-inferred log_2_ fold change of gene expression across layers. Points with absolute value greater than 1 are colored as pink, otherwise gray. (**K**) Gene expression profiles of POLARIS-identified LDE genes in the cancer in situ layer.

As a starting point for assessing POLARIS’ ability to infer LDE genes, we performed simulations where we know the truth. Following the same simulation framework used above to evaluate deconvolution efficiency, we evaluated the layer-specific location parameters. Again, because cellular composition is simulated from the same distribution across spots regardless of layer status, observed gene expression variation can only be attributed to truly differential expression patterns across layers (Fig. 2, A and B). Consequently, genes could be classified into three categories: layer 1–enriching genes, layer 2–enriching genes, and genes with similar expression levels across layers (Figs. 2B and 3A). We applied POLARIS to perform deconvolution and simultaneously perform the permutation test and calculate the log_2_ fold change in mean expression, layer 1 over layer 2. POLARIS successfully identified genes that have different expression profiles across layers (Fig. 3B). The predicted log_2_ fold change well captures the true log_2_ fold change (Fig. 3C) when all the genes have layer-specific gene expression profiles. In this particular simulation, we generated the genes such that all of them have layer-specific expression profiles, although the expression difference between layers of some genes could be small (detailed in the Supplementary Materials). To investigate the impact of the proportion of LDE genes on POLARIS performance, we further conducted simulations using the same setting but varying the proportion of genes with layer-specific expression profiles (fig. S2). As the proportion of LDE genes increases from 0 to 1, Uniform Manifold Approximation and Projection (UMAP) representations of cells become more separated by layers. It is difficult, however, to identify the subtypes of cells based on the UMAP representation, even with prior knowledge of the layer information when the proportion of LDE genes is <0.5, thus failing to capture heterogeneity across layers (fig. S2A). POLARIS well controlled the type I error and accurately estimated the true log_2_ fold change regardless of the proportion of LDE genes (fig. S2B). POLARIS also shows satisfactory performance on a sparse simulation setting (fig. S3). Note that layer-specific parameters are inferred only from the ST data because we do not have layer information from the scRNA-seq reference.

Encouraged by POLARIS’s performance in simulated data, we proceeded to further test POLARIS’s capability to detect LDE genes using real datasets. We began with the developing human heart single-cell resolution ISS ST data. We created pseudo-spots from the dataset and applied POLARIS to the pseudo-spots, feeding as input spot-level gene expression information along with layer information inferred from BayesSpace. LDE genes detected by POLARIS have differential patterns of gene expression in different layers (Fig. 3, D and E). Although cell type affects gene expression, layer status also plays an essential role in shaping the spot-level expression profile. Furthermore, these layer-specific changes are shared by many cell types. For example, the expression of *EBF2* in most cell types is highest in layer 3 compared to the other three layers (Fig. 3E). POLARIS, by making accurate inference of gene expression profiles in different cell types, has successfully captured the LDE gene and has recovered the layer-specific variation present in most cell types. We further explore the links between LDE genes and biological functions of each layer. For example, among the LDE genes, *TCF21* has the lowest mean gene expression in layer 3 (Fig. 3, D and E) where fibroblast-like cells are enriched (Fig. 2, J and K). Transcription factor 21 (TCF21) encoded by *TCF21* plays a crucial role in regulating cell differentiation and cell fate determination through epithelial-mesenchymal transformations during cardiac development. More specifically, TCF21 has been reported to be capable of promoting the development of cardiac fibroblasts and inhibiting differentiation of epicardial cells into vascular smooth muscle cells (*37*), consistent with our observed down-regulation in layer 3.

We further applied POLARIS on a more complex tissue: breast cancer samples. There are several subtypes of breast cancer, among which the HER2+ subtype is characterized by the increased expression of *ERBB2* (also known as *HER2*) in tumor cells (*38*). We obtained ST data of HER2+ tumors from eight individuals (patients A to H) generated through the Spatial Transcriptomics platform (*3*, *38*). Each of the eight patients provided multiple slides, but only one slide from each patient was pathologist annotated. Annotations mark areas with one of the following six labels: in situ cancer [noninvasive ductal carcinoma in situ (DCIS)], invasive breast cancer (IBC), adipose tissue, immune infiltrate, breast glands, or connective tissue (undetermined spots were not used in the analysis) (*38*). Here, we highlight the results of two slides with both IBC and DCIS regions (results from the A1 slide in Fig. 3, F to K, and fig. S5 and results from the G2 slide in figs. S4 and S5). We applied POLARIS on the pathologist-annotated ST data using an external scRNA-seq reference (*39*). POLARIS made reasonable inference regarding cell composition on slide A1 (Fig. 3F). For example, cancer epithelial cells are inferred to be enriched in the DCIS and IBC areas. POLARIS also identified several LDE genes in the DCIS area (Fig. 3, I and J). These LDE genes, including *S100A14*, *MUC1*, *PITX1*, and *ERBB2*, are mainly expressed in cancer epithelial cells (fig. S6). In general, genes that are primarily expressed in cancer epithelial cells are enriched in either the DCIS or the IBC region (Fig. 3, H to K), which is expected because these two regions have similarly high proportions of cancer epithelial cells. The LDE genes identified by POLARIS successfully capture cancer epithelial cell–specific genes which differentially expressed in the two regions (Fig. 3, H to K). For example, in the DCIS region, all POLARIS-identified LDE genes except *ERBB2* have a positive log_2_ fold change estimate (Fig. 3J), consistent with expression patterns shown in Fig. 3K. The observed down-regulation of *ERBB2* in the DCIS area most likely reflects an up-regulation of *ERBB2* elsewhere. As shown in Fig. 3F, most of the slide A1 is the invasive cancer area. Cancer epithelial cells in the invasive cancer area presumably invade other areas, resulting in an increased *ERBB2* expression in all other pathologist-identified areas except the DCIS, and these invaded areas also exhibit an increase in the proportion of cancer epithelial cells (Fig. 3, H and I, and fig. S4F). ERBB2, the protein encoded by *ERBB2*, plays an important role in breast cancer. The overexpression of *ERBB2* disrupts normal cell-control mechanisms, gives rise to aggressive tumor cells, and leads to increased breast cancer metastasis (*40*–*45*). When applied to slide G2, *ERBB2* is shown to be up-regulated in the DCIS area (fig. S4, E and G). Together, these observations reflect the heterogeneity of the samples and are consistent with the literature that *ERBB2* is overexpressed in 30 to 35% of DCIS, while *ERBB2* is only expressed in 15 to 25% of IBC (*46*–*49*). POLARIS reveals this heterogeneity and complexity by showing differential gene expression profiles between DCIS and IBC regions on the same slide and by revealing differential patterns across slides from the same patient as well as across patients. In addition to *ERBB2*, other LDE genes identified by POLARIS and the proteins encoded by those genes also play important roles in breast cancer. For example, copy number amplification of *S100A14*, significantly correlated with the increased *S100A14* mRNA expression, is present in 5.4 to 20.7% of primary breast cancer patients and in approximately 26.1% of metastatic breast cancer patients (*50*). For another example, *CXXC5* overexpression has been observed to be associated with a poor prognosis for estrogen receptor–positive (ER+) breast cancer (*51*).

### Polaris enables prediction purely from histology image

After demonstrating that even a single-color histology image is able to generate high-accuracy deconvolution inference in the developing human heart tissue, we continued to apply POLARIS to other spot-level ST data with H&E-stained images. We used the mouse primary somatosensory cortex area (SSp) data generated from the 10x Visium platform (*52*). Similar to the mouse VISp region, mouse SSp is also an area in the mouse cortex with well-defined anatomical and functional structure. Specifically, the glutamatergic neuron types exhibit clear layered patterns (*6*, *33*, *53*). Using an independent scRNA-seq from similar SSp regions (*33*) as the external reference, POLARIS trained an image network using four SSp ST slides. Each slide was clustered into six groups using BayesSpace (fig. S7). POLARIS successfully captures expected patterns of glutamatergic composition and reveals layer structure consistent with data from the Allen Brain Atlas (*34*).

**Fig. 4.**
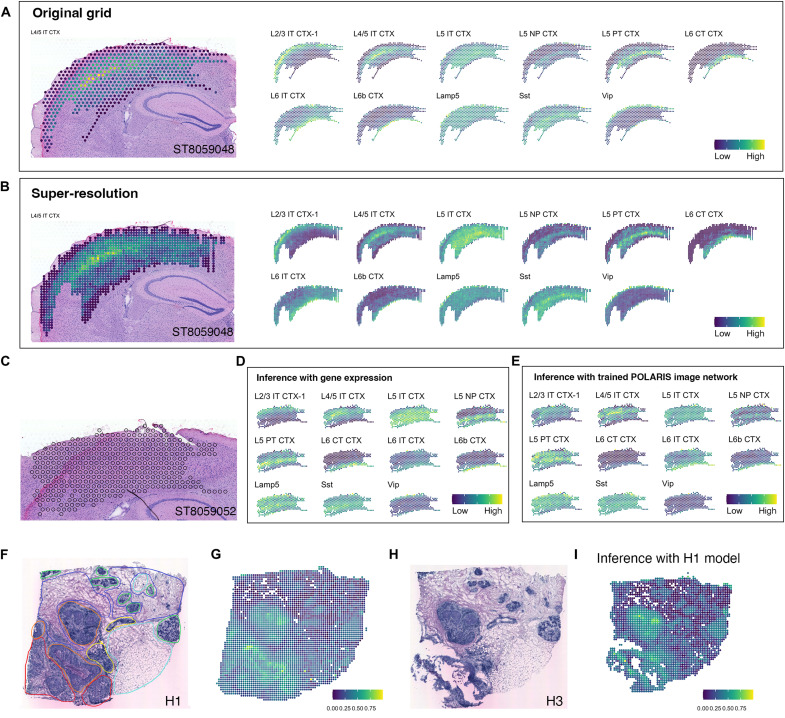
POLARIS achieves super-resolution cell composition inference when using histology image as input. On mouse SSp 10x Visium slide ST8059048: (**A**) Original grid and POLARIS-inferred cell composition. (**B**) Super-resolution cell composition inferred using a POLARIS-trained image network. Points in (A) and (B) are colored by the corresponding cell proportion (from blue to yellow corresponds to low to high). On slide ST8059052: (**C**) Original grid of the slide. (**D**) POLARIS-inferred cell composition inference using spot-level gene expression and histology image. (**E**) Inferred cell composition using a POLARIS-trained image network (trained on the other four slides), based on histology image only. Similarly, points in (D) and (E) are colored by the corresponding cell proportion (from blue to yellow corresponds to low to high). On HER2+ breast cancer: (**F**) Pathologist’s annotation of slide H1. (**G**) Super-resolution inference of H1 using POLARIS image network trained on H1. (**H**) Histology image of slide H3. (**I**) Super-resolution inference of H3 using POLARIS image network, again trained on H1. The points in (G) and (I) are colored by the cancer epithelial cell proportion. CTX, isocortex; IT, intratelencephalic; PT, pyramidal tract; NP, near-projecting.

Spot-level ST technologies cannot measure every part of a tissue slide. As shown in Fig. 4A, gene expression levels are not available for any region outside the measured spots (Fig. 4A). POLARIS, through its trained image network, can determine cell type composition using the image of unmeasured areas. Specifically, POLARIS-trained image network can be applied to cropped images of the same size as the original grid in training. Sliding across the entire histological image of the SSp slide and applying the POLARIS-trained image network to the image of each sliding window, one can obtain super-resolution inference, encompassing areas not initially covered by spatial spots. This super-resolution inference empowers us to gain finer details of the layered structure (Fig. 4B). Following the training of POLARIS’s image network, we applied it to a new SSp slide to test whether the trained image network is able to make reasonable inference on images from independent slides (Fig. 4C). We compared cell compositions inferred using the image network trained by POLARIS from other slides, with those inferred using the new slide’s own gene expression and histology image on measured spots (Fig. 4, D and E, and fig. S8, A and B). Although the two sets of inference show differences, results obtained solely from POLARIS’s pretrained image network still show strong correlation with the inference results using its own image and gene expression, especially in glutamatergic neurons (e.g., the Pearson’s correlation for L6b CTX is 0.8). POLARIS-trained image network successfully recovers the layered structure in most cell types, suggesting that the approach could be applied to new histology slides as long as we have a network pretrained by POLARIS using ST data from similar regions.

POLARIS-trained image network was further examined using breast cancer data. We again used pathologist-annotated ST data and the external scRNA-seq from Wu and colleagues as reference to perform deconvolution on slide H1 (Fig. 4F) (*38*, *39*). Afterward, we used the image network trained by POLARIS from slide H1 to enhance resolution and obtain super-resolution cell composition inference on the H1 slide itself (Fig. 4G). The inferred cell compositions are consistent with those in other slides where cancer epithelial cells are enriched in the DCIS and IBC regions. It appears that cancer epithelial cell proportion is able to accurately capture the cancerous areas. For example, spots with >0.4 proportion of cancer epithelial cells are enriched within regions labeled as DCIS and IBC by pathologists (fig. S8E). We then further applied the POLARIS image network trained on H1 to two other slides of the same patients (H2 and H3), which have no pathologist’s annotation (Fig. 4, H and I, and fig. S8, C to G). By closely examining the histology images, it is evident that regions characterized by high proportions of cancer epithelial cells are primarily cancerous areas. Our results therefore suggest that POLARIS enables a new method of registering histology images to different anatomical/functional regions, e.g., cancerous areas in this analysis. By examining cell composition in each spot, we are able to group the spots into layers. In summary, with a trained POLARIS image network, we could obtain super-resolution cell composition inference, which reveals finer layer structure of a tissue.

POLARIS allows cell composition inference on new histology images without gene expression and, consequently, is able to identify anatomical and functional regions. Compared to existing supervised classification methods for registering histology image tiles to different regions or layers, which require annotated layer information, POLARIS is unsupervised in terms of histology information and does not require pathologist annotation. Instead, POLARIS relies on preknowledge about the relationship between the targeted layer and the cell composition.

## DISCUSSION

ST technologies are rapidly evolving. In the near future, we expect to be able to measure gene expression levels at single-cell resolution and of all genes in the transcriptome. Now, spot-level resolution ST technologies such as the Visium and Spatial Transcriptomes still have their advantages in terms of throughput (in terms of both number of genes measured and number of spatial spots examined) and the ability to obtain high-resolution H&E-stained images. Because of their advantages, researchers are generating a deluge of these data. It is, however, imperative to perform cell type deconvolution at each spot to mitigate or eliminate potential confounding caused by differential cell composition across spots. Despite numerous methods developed for ST deconvolution, two pieces of information have been underused. First, often there is a layer structure or at least areas reflecting different anatomical or functional regions in an ST slide. Second, histological images, carrying information complementary to spot-level gene expression profiles, have not been fully explored in their value for cell type decomposition. In this work, we present POLARIS, a unified framework that leverages layer structure information and/or histological images, for cell type deconvolution both at spots with expression measurement and in regions with only image information, as well as for the revelation of LDE genes.

We demonstrate the performance of POLARIS on simulation and real datasets including developing human heart, mouse cortex VISp and SSp region, and human HER2+ breast cancer samples. POLARIS robustly achieves best or close to best deconvolution performance compared to other state-of-the-art methods. POLARIS’s inference on spot-level ST data reveals layered structures that are consistent with gene expression profiles, histological images, and known/established anatomical/functional layers/regions in the corresponding tissue samples.

Equally if not more importantly, POLARIS accurately infers layer-specific expression profiles across different cell types, which leads to the identification of LDE genes. We demonstrate POLARIS’s power to identify LDE genes using simulation data as well as the single-cell resolution ST data from the developing human heart, where we have knowledge regarding the true LDE genes. The LDE genes identified by POLARIS in developing human heart data exhibit different gene expression profiles across layers, beyond what can be attributed to differential cell type compositions. Last, applying POLARIS to ST data from breast cancer patients, we found that POLARIS-identified LDE genes reveal complex heterogeneous across-layer/region differential expression across samples and/or patients. The detected LDE genes are consistent with established knowledge regarding breast cancer pathology, including metastasis and prognosis, but offer more granular sample-level and patient-level information that can potentially empower personalized diagnosis and treatment. POLARIS assumes that the layer-specific shift parameter is shared across cell types. This assumption is based on observations in single-cell resolution ST data (e.g., heart ISS data; Fig. 3E). In addition, because of the model identification problem, we chose to go with a parsimonious model and therefore did not implement a layer shift specific to each cell type. Considering that we are inferring both the cell type proportion and the layer-specific shift at the same time, modeling a layer shift specific to each cell type will very likely cause the model to confuse the proportion and shift, which will result in poor and unstable estimation of both parameters. We consider this as a limitation of our model, which will likely to be resolved in future efforts.

Another key feature of POLARIS is its ability to leverage image data. In the ST deconvolution field, gene expression itself has proved its ability to infer cell composition. Imaging information, however, has been underused. Recent work (*18*, *19*) showed the potential of histology images accompanying ST data. We believe that histology images can be further leveraged for ST inference. For example, histology images alone are widely used to segment cells with deep learning models (*13*, *14*). POLARIS can take an accompanying image as input to train an image network and use a pretrained image network on a completely new image. Our pretrained POLARIS image network offers a novel method for tissue registration, which extracts and reveals tissue anatomical or functional structures either from the histological image alone or jointly with gene expression. The major barriers that prevent the full potential of integrating histological images with ST data include quality of the co-registered image and, most importantly, the absence of pathologist annotations. To accomplish the task with the currently available data, POLARIS incorporates training of the image network into the inference of cell composition. Instead of training a model with inferred cell composition as the goal and using MSE as the loss function, our intention is to use histology images to help with the estimation of cell composition under the rationale that spots with similar histological images and similar neighborhoods tend to share similar cell composition. Nevertheless, we fail to demonstrate that the image improves deconvolution performance due to the limitation of the current data: Single cell–level resolution ST data only provide DAPI-stained images, which only comprise one color panel, while spot-level ST data have no gold standard truth. Despite these limitations to quantify the performance, POLARIS with image input still achieved high accuracy among the state-of-the-art methods in single-cell resolution ST data, and in spot-level ST data, cell type composition inferred by POLARIS agreed with single-cell level data and the expected biological layers (e.g., glutamatergic neurons in the mouse cortex and cancer epithelial cells in the breast cancer slides). POLARIS introduces a novel approach for inferring cell composition purely from histological images that has not previously been explored by ST deconvolution. We believe that the versatile ability of POLARIS to incorporate histological images to elucidate layer-specific gene expression patterns will empower discoveries in spatial biology.

## MATERIALS AND METHODS

### POLARIS inference algorithm

We use *g* for gene index, *c* for cell index, *z* for cell type index, and *L* for layer index. *Z_c_* is the cell type of cell *c*, *L_c_* is the layer of cell *c*, and *L_s_* is the layer of spot *s*. We assume that in scRNA-seq reference dataset, *X_cg_*, expression count of gene *g* in cell *c* follows the following negative binomial distribution

$$X_{cg}∼\mathrm{NB}[S_{c}\;\mathrm{Softplus}(\theta_{gz_{c}}+T_{L_{c}g}),P_{g}]$$

Softplus(*x*) = log[1 + exp(*x*)] is used to guarantee that *R_gz_c__* = Softplus(θ*_gz_c__*+*T_L_c_g_*) > 0. *P_g_* = Sigmoid(*o_g_*), where $\mathrm{Sigmoid}(x)=\frac{1}{1+\exp(-x)}$ is the sigmoid function. *S_c_* is the library size of cell *c*, and *Z_c_* is the cell type of cell *c*. NB is the negative binomial distribution. θ*_gz_c__* is the mean location parameter shared across layers. To capture the gene expression variation between layers, we introduce a new parameter *T_Lg_*, a mean shift parameter for layer *L* and gene *g*. We assume that for a gene, all cell types share the same shift parameter across layers. We further assume that *T_Lg_* ∼ *N*(0,1). However, in real world, we do not have the layer information in the scRNA-seq reference data. We make the following assumption

$$X_{cg}∼\mathrm{NB}[S_{c}\;\mathrm{Softplus}(\theta_{gz_{c}}),P_{g}]$$

when inferring the parameters in the reference data. Estimates for the parameters are then obtained by finding the maximum likelihood estimate, given the provided scRNA-seq reference data via the gradient-based optimization using the PyTorch library in Python.

In the ST data, we make similar distributional assumption. Specifically, we assume that *X_scg_*, expression count of gene *g* in cell *c* in spot *s*, follows the following negative binomial distribution

$$X_{scg}∼\mathrm{NB}[\beta_{g}\mathrm{Softplus}(\theta_{gz_{c}}+T_{L_{c}g}),P_{g}]$$

β*_g_* is the parameter measuring technical bias between ST and scRNA-seq reference for gene *g*. Note that the bias parameter is gene specific. Considering the additive property of negative binomial distribution and summing across all single cells within spot *s*, the resulting distribution of gene *g* in spot *t* also follows a negative binomial distribution

$$X_{sg}∼\mathrm{NB}\left[\sum_{z=1}^{Z}\beta_{g}\mathrm{Softplus}(\theta_{gz}+T_{L_{s}g})n_{sz},P_{g}\right]$$

*Z* is the total number of cell types, and *n_sz_* is the number of cells of type *z* in spot *s*. All the parameters are inferred using MAP estimation, with $\hat{\theta_{g}},\hat{P_{g}}$ obtained from scRNA-seq reference. When image data are used in the model, we incorporate a POLARIS image network to infer the cell type composition. POLARIS image network takes MAE-extracted features as input, and the output is the cell composition. The image network is trained using MAP, which means that the loss function is the negative posterior likelihood. Last, cell type proportion can be calculated as $v_{sz}=\frac{n_{sz}}{\sum n_{sz}}$.

### Image feature extraction using MAE

The MAE codes are obtained from https://github.com/facebookresearch/mae (version 11 January 2022). We only use the pretrained encoder part to extract the image features (*32*). We used mae_visualize_vit_large_ganloss.pth as the pretrained model. We used MAE to extract image features for the spot image and spot neighborhood image. The spot image is a r*r square covering the spot. *r* is defined specific to the dataset used (details about the used *r* could be found in the source code). The neighborhood image is defined as a 3r*3*r* square sharing the same center point as the spot image. The two MAE-extracted 1024 length vectors are combined as the image input of the spot. The POLARIS image network combines two levels of fully connected layers (2048 to 512, 512 to the number of cell types). θ*_gz_* and *T_L_s_g_* are fixed during the training of image network.

### Simulation

We begin by generating a single-cell reference. A total of 100 genes are simulated in six cell types. The number of cells in each cell type is simulated from *N*(500,100^2^) and rounded to the nearest integer. For each cell, we simulate the layer from Binomial(0.3), where we treat a simulated value of 0 as layer 1 and value of 1 as layer 2. We then simulate the gene expression of gene *g* in cell *c* from NB[Softplus(θ*_gz_c__* + *T_L_c_g_*), *P_g_*], where NB denotes the negative binomial distribution. *Z_c_* represents the cell type of cell *c*, and *L_c_* represents the layer of cell *c*. In the first simulation, both θ*_gz_c__* and *T_Lg_* are simulated from *N*(0,1) and *P_g_* is simulated from Uniform(0.2,0.8). In the sparse simulation, θ*_gz_c__* are simulated from *N*( − 2,1).Note that *T_Lg_* represents the layer-specific gene expression profile, thus allowing expression profiles to vary across layers, even for the same gene *g* and cell type. We then construct pseudo-spots by randomly selecting cells in layer 1 and layer 2 with replacement from single cells simulated above. In each spot, the number of cells is determined by sampling a random number from Uniform (10,16). We simulated 50 spots in layer 1 and 150 spots in layer 2. For simulation with different proportion of genes with layer-specific expression profiles, we define the proportion as *m*. Then, *T_Lg_* for 100^*^(1 − *m*)% genes are set as 0, while *T_Lg_* for the rest 100^*^m% genes are simulated from *N*(0,1).

### Calculate between-layer fold change

To identify LDE genes, we compare the gene expression profile across layers by comparing layer-specific location parameters

$$\mathrm{Softplus}(\theta_{gz}+T_{Lg})$$

Specifically, the fold change of gene *g* in layer *L* compared to other layers in cell type *z* is calculated as

$$F_{Lg}=\mathrm{Softplus}(\theta_{gz}+T_{Lg})/\left[\sum_{L\prime \neq L}\mathrm{Softplus}(\theta_{gz}+T_{L\prime g})/(S-1)\right]$$

where *S* is the total number of layers. Then, we take the maximum fold change across cell types to quantify the across-layer fold change of gene *g*.

### LDE gene identification

We perform permutation test to identify LDE genes. With a given layer annotation Layer^obs^, we perform cell type deconvolution and infer $T_{Lg}^{\mathrm{obs}}$ and $F_{Lg}^{\mathrm{obs}}$. Then, we randomize the layer annotation for *N* times and generate pseudo-layer annotation Layer^sim1^, …, Layer^sim*N*^. Consequently, we are able to infer $T_{Lg}^{\mathrm{sim}1},\ldots ,T_{Lg}^{\mathrm{sim}N}$. Then, the *P* value of gene *g* in layer *L* is defined as

$$P\;\mathrm{value}=\frac{\#|T_{Lg}^{\mathrm{sim}}|>|T_{Lg}^{\mathrm{obs}}|}{N}$$

Genes with *P* value $<\frac{0.05}{\text{Total number of genes}}$ (Bonferroni correction) and $|\log_{2}F_{Lg}^{\mathrm{obs}}|>1$ are considered as the LDE genes in layer *L* (marked as pink in the figures). We used *N* = 10,000 (permutation times) in all the analysis. For the LDE analysis in the article, we only used gene expression (i.e., histological images not used) in the deconvolution process in the permutation test.

### Data preprocessing

For the STARmap data, we only keep genes that are present in at least 2% of spots.

In the breast cancer analysis, we only keep HER2^+^ subtype ST data for deconvolution evaluation. Similarly, we keep only the HER2^+^ patients’ scRNA-seq data as reference. For scRNA-seq reference, genes expressed in at least three cells and cells expressing at least 200 genes are kept. Similarly for ST data, genes expressed in at least three spots and spots expressing at least 100 genes are kept. Only genes that exist in both scRNA-seq and ST data are used in further analysis. Top 2000 highly variable genes (HVGs) are used. The gene subsettings are accomplished using the R package Seurat (54). HVGs are selected using feature variance calculated by the FindVariableFeatures function with default settings. To reduce the effect of excessive zeros, we recommend removing genes with an expression level of less than 2%. For the heart ISS DAPI-stained image, we reverse the color and enhance the contrast using ImageEnhance function in the Pillow package (55).

### Comparing to other state-of-the-art methods

We compared the performance of POLARIS with several state-of-the-art deconvolution methods developed for ST data. We included RCTD and stereoscopes because of their top performance in the probabilistic-based model. Our inclusion of DestVI was based on the fact that it claimed to identify a continuous state that goes beyond discrete cell types, which we consider to be similar to our objective. CARD was included because it is capable of inferring the cell type composition in areas that are not directly measured. Because SPOTlight is the top performer among the NMF+NNLS techniques, we also included it in our analysis.

We followed the instructions of each method on their corresponding website. Among all the methods, only RCTD has a built-in gene filtering method, where only genes with normalized gene expression ≥ 0.0002 are included, and it selects cell type marker genes based on a log fold change threshold of 0.75 (*28*). We used the default parameters of RCTD and ran RCTD in full mode. Only selected cell type marker genes were fed into RCTD. For all other methods, we used all genes without any further filtering from the preprocessed data described in the previous section.

### Tissue detection

To use POLARIS-trained image network, we need to automatically detect the tissue section from a histological image. We used the filter_entropy function in https://github.com/CODAIT/deep-histopath (version 7 March 2019). We used entropy, which measures tissue complexity, to detect the percentage of tissue in each spot. Areas such as the slide background are less complex than the tissue area. We used the default threshold of 5 in the analysis. Pixels with an entropy value greater than 5 are counted as tissue regions. Spots with greater than 5% tissue area are kept in the super-resolution composition inference.

## Notes

View/request a protocol for this paper from *Bio-protocol*.

## References

[1] C. Xia, J. Fan, G. Emanuel, J. Hao, X. Zhuang, Spatial transcriptome profiling by MERFISH reveals subcellular RNA compartmentalization and cell cycle-dependent gene expression. Proc. Natl. Acad. Sci. U.S.A. 116, 19490–19499 (2019). 31501331 10.1073/pnas.1912459116 PMC6765259

[2] X. Wang, W. E. Allen, M. A. Wright, E. L. Sylwestrak, N. Samusik, S. Vesuna, K. Evans, C. Liu, C. Ramakrishnan, J. Liu, G. P. Nolan, F. A. Bava, K. Deisseroth, Three-dimensional intact-tissue sequencing of single-cell transcriptional states. Science 361, (2018). 10.1126/science.aat5691 PMC6339868 29930089

[3] P. L. Ståhl Patrik, F. Salmén, S. Vickovic, A. Lundmark, J. F. Navarro, J. Magnusson, S. Giacomello, M. Asp, J. O. Westholm, M. Huss, A. Mollbrink, S. Linnarsson, S. Codeluppi, Å. Borg, F. Pontén, P. I. Costea, P. Sahlén, J. Mulder, O. Bergmann, J. Lundeberg, J. Frisén, Visualization and analysis of gene expression in tissue sections by spatial transcriptomics. Science 353, 78–82 (2016). 27365449 10.1126/science.aaf2403

[4] R. R. Stickels, E. Murray, P. Kumar, J. Li, J. L. Marshall, D. J. di Bella, P. Arlotta, E. Z. Macosko, F. Chen, Highly sensitive spatial transcriptomics at near-cellular resolution with Slide-seqV2. Nat. Biotechnol. 39, 313–319 (2021). 33288904 10.1038/s41587-020-0739-1 PMC8606189

[5] C.-H. L. Eng, M. Lawson, Q. Zhu, R. Dries, N. Koulena, Y. Takei, J. Yun, C. Cronin, C. Karp, G. C. Yuan, L. Cai, Transcriptome-scale super-resolved imaging in tissues by RNA seqFISH+. Nature 568, 235–239 (2019). 30911168 10.1038/s41586-019-1049-y PMC6544023

[6] J. Chen, W. Liu, T. Luo, Z. Yu, M. Jiang, J. Wen, G. P. Gupta, P. Giusti, H. Zhu, Y. Yang, Y. Li, A comprehensive comparison on cell-type composition inference for spatial transcriptomics data. Brief. Bioinform. 23, bbac245 (2022). 35753702 10.1093/bib/bbac245 PMC9294426

[7] L. Moses, L. Pachter, Museum of spatial transcriptomics. Nat. Methods 19, 534–546 (2022). 35273392 10.1038/s41592-022-01409-2

[8] A. Rao, D. Barkley, G. S. França, I. Yanai, Exploring tissue architecture using spatial transcriptomics. Nature 596, 211–220 (2021). 34381231 10.1038/s41586-021-03634-9 PMC8475179

[9] S. Bae, H. Choi, D. S. Lee, Discovery of molecular features underlying the morphological landscape by integrating spatial transcriptomic data with deep features of tissue images. Nucleic Acids Res. 49, e55 (2021). 33619564 10.1093/nar/gkab095 PMC8191797

[10] Y. Zong, T. Yu, X. Wang, Y. Wang, Z. Hu, V. Y. Li, conST: An interpretable multi-modal contrastive learning framework for spatial transcriptomics. bioRxiv 2022.01.14.476408 [Preprint]. 17 January 2022. 10.1101/2022.01.14.476408.

[11] J. Hu, X. Li, K. Coleman, A. Schroeder, N. Ma, D. J. Irwin, E. B. Lee, R. T. Shinohara, M. Li, SpaGCN: Integrating gene expression, spatial location and histology to identify spatial domains and spatially variable genes by graph convolutional network. Nat. Methods 18, 1342–1351 (2021). 34711970 10.1038/s41592-021-01255-8

[12] B. He, L. Bergenstråhle, L. Stenbeck, A. Abid, A. Andersson, Å. Borg, J. Maaskola, J. Lundeberg, J. Zou, Integrating spatial gene expression and breast tumour morphology via deep learning. Nat. Biomed. Eng. 4, 827–834 (2020). 32572199 10.1038/s41551-020-0578-x

[13] J. Xu, L. Xiang, Q. Liu, H. Gilmore, J. Wu, J. Tang, A. Madabhushi, Stacked sparse autoencoder (SSAE) for nuclei detection on breast cancer histopathology images. IEEE Trans. Med. Imaging 35, 119–130 (2016). 26208307 10.1109/TMI.2015.2458702 PMC4729702

[14] M. Tofighi, T. Guo, J. K. P. Vanamala, V. Monga, Deep networks with shape priors for nucleus detection, in *2018 25th IEEE International Conference on Image Processing (ICIP)* (IEEE, 2018).

[15] A. C. Daly, K. J. Geras, R. Bonneau, A convolutional neural network for common coordinate registration of high-resolution histology images. Bioinformatics 37, 4216–4226 (2021). 34128955 10.1093/bioinformatics/btab447 PMC9502165

[16] N. Coudray, P. S. Ocampo, T. Sakellaropoulos, N. Narula, M. Snuderl, D. Fenyö, A. L. Moreira, N. Razavian, A. Tsirigos, Classification and mutation prediction from non–small cell lung cancer histopathology images using deep learning. Nat. Med. 24, 1559–1567 (2018). 30224757 10.1038/s41591-018-0177-5 PMC9847512

[17] H. Liao, Y. Long, R. Han, W. Wang, L. Xu, M. Liao, Z. Zhang, Z. Wu, X. Shang, X. Li, J. Peng, K. Yuan, Y. Zeng, Deep learning-based classification and mutation prediction from histopathological images of hepatocellular carcinoma. Clin. Transl. Med. 10, e102 (2020). 32536036 10.1002/ctm2.102 PMC7403820

[18] T. Biancalani, G. Scalia, L. Buffoni, R. Avasthi, Z. Lu, A. Sanger, N. Tokcan, C. R. Vanderburg, Å. Segerstolpe, M. Zhang, I. Avraham-Davidi, S. Vickovic, M. Nitzan, S. Ma, A. Subramanian, M. Lipinski, J. Buenrostro, N. B. Brown, D. Fanelli, X. Zhuang, E. Z. Macosko, A. Regev, Deep learning and alignment of spatially resolved single-cell transcriptomes with Tangram. Nat. Methods 18, 1352–1362 (2021). 34711971 10.1038/s41592-021-01264-7 PMC8566243

[19] A. Zubair, R. H. Chapple, S. Natarajan, W. C. Wright, M. Pan, H.-M. Lee, H. Tillman, J. Easton, P. Geeleher, Cell type identification in spatial transcriptomics data can be improved by leveraging cell-type-informative paired tissue images using a Bayesian probabilistic model. Nucleic Acids Res. 50, e80 (2022). 35536287 10.1093/nar/gkac320 PMC9371936

[20] J. Zhu, S. Sun, X. Zhou, SPARK-X: Non-parametric modeling enables scalable and robust detection of spatial expression patterns for large spatial transcriptomic studies. Genome Biol. 22, 184 (2021). 34154649 10.1186/s13059-021-02404-0 PMC8218388

[21] V. Svensson, S. A. Teichmann, O. Stegle, SpatialDE: Identification of spatially variable genes. Nat. Methods 15, 343–346 (2018). 29553579 10.1038/nmeth.4636 PMC6350895

[22] E. Zhao, M. R. Stone, X. Ren, J. Guenthoer, K. S. Smythe, T. Pulliam, S. R. Williams, C. R. Uytingco, S. E. B. Taylor, P. Nghiem, J. H. Bielas, R. Gottardo, Spatial transcriptomics at subspot resolution with BayesSpace. Nat. Biotechnol. 39, 1375–1384 (2021). 34083791 10.1038/s41587-021-00935-2 PMC8763026

[23] Q. Zhu, S. Shah, R. Dries, L. Cai, G. C. Yuan, Identification of spatially associated subpopulations by combining scRNAseq and sequential fluorescence in situ hybridization data. Nat. Biotechnol. 36, 1183–1190 (2018). 10.1038/nbt.4260 PMC6488461 30371680

[24] B. F. Miller, F. Huang, L. Atta, A. Sahoo, J. Fan, Reference-free cell type deconvolution of multi-cellular pixel-resolution spatially resolved transcriptomics data. Nat. Commun. 13, 2339 (2022). 35487922 10.1038/s41467-022-30033-z PMC9055051

[25] M. N. Bernstein, Z. Ni, A. Prasad, J. Brown, C. Mohanty, R. Stewart, M. A. Newton, C. Kendziorski, SpatialCorr identifies gene sets with spatially varying correlation structure. Cell Rep. Methods 2, 100369 (2022). 36590683 10.1016/j.crmeth.2022.100369 PMC9795364

[26] Y. Ma, X. Zhou, Spatially informed cell-type deconvolution for spatial transcriptomics. Nat. Biotechnol. 40, 1349–1359 (2022). 35501392 10.1038/s41587-022-01273-7 PMC9464662

[27] A. Andersson, J. Bergenstråhle, M. Asp, L. Bergenstråhle, A. Jurek, J. Fernández Navarro, J. Lundeberg, Single-cell and spatial transcriptomics enables probabilistic inference of cell type topography. Commun. Biol. 3, 565 (2020). 33037292 10.1038/s42003-020-01247-y PMC7547664

[28] D. M. Cable, E. Murray, L. S. Zou, A. Goeva, E. Z. Macosko, F. Chen, R. A. Irizarry, Robust decomposition of cell type mixtures in spatial transcriptomics. Nat. Biotechnol. 40, 517–526 (2022). 33603203 10.1038/s41587-021-00830-w PMC8606190

[29] R. Lopez, B. Li, H. Keren-Shaul, P. Boyeau, M. Kedmi, D. Pilzer, A. Jelinski, I. Yofe, E. David, A. Wagner, C. Ergen, Y. Addadi, O. Golani, F. Ronchese, M. I. Jordan, I. Amit, N. Yosef, DestVI identifies continuums of cell types in spatial transcriptomics data. Nat. Biotechnol. 40, 1360–1369 (2022). 35449415 10.1038/s41587-022-01272-8 PMC9756396

[30] M. Elosua-Bayes, P. Nieto, E. Mereu, I. Gut, H. Heyn, SPOTlight: Seeded NMF regression to deconvolute spatial transcriptomics spots with single-cell transcriptomes. Nucleic Acids Res. 49, e50 (2021). 33544846 10.1093/nar/gkab043 PMC8136778

[31] B. Li, W. Zhang, C. Guo, H. Xu, L. Li, M. Fang, Y. Hu, X. Zhang, X. Yao, M. Tang, K. Liu, X. Zhao, J. Lin, L. Cheng, F. Chen, T. Xue, K. Qu, Benchmarking spatial and single-cell transcriptomics integration methods for transcript distribution prediction and cell type deconvolution. Nat. Methods 19, 662–670 (2022). 35577954 10.1038/s41592-022-01480-9

[32] K. He, X. Chen, S. Xie, Y. Li, P. Dollár, R. Girshick, Masked autoencoders are scalable vision learners. arXiv:2111.06377 [cs.CV] (11 November 2021).

[33] Z. Yao, C. T. J. van Velthoven, T. N. Nguyen, J. Goldy, A. E. Sedeno-Cortes, F. Baftizadeh, D. Bertagnolli, T. Casper, M. Chiang, K. Crichton, S. L. Ding, O. Fong, E. Garren, A. Glandon, N. W. Gouwens, J. Gray, L. T. Graybuck, M. J. Hawrylycz, D. Hirschstein, M. Kroll, K. Lathia, C. Lee, B. Levi, D. McMillen, S. Mok, T. Pham, Q. Ren, C. Rimorin, N. Shapovalova, J. Sulc, S. M. Sunkin, M. Tieu, A. Torkelson, H. Tung, K. Ward, N. Dee, K. A. Smith, B. Tasic, H. Zeng, A taxonomy of transcriptomic cell types across the isocortex and hippocampal formation. Cell 184, 3222–3241.e26 (2021). 34004146 10.1016/j.cell.2021.04.021 PMC8195859

[34] Allen Reference Atlas—Mouse Brain [brain atlas]; atlas.brain-map.org.

[35] M. Asp, S. Giacomello, L. Larsson, C. Wu, D. Fürth, X. Qian, E. Wärdell, J. Custodio, J. Reimegård, F. Salmén, C. Österholm, P. L. Ståhl, E. Sundström, E. Åkesson, O. Bergmann, M. Bienko, A. Månsson-Broberg, M. Nilsson, C. Sylvén, J. Lundeberg, A spatiotemporal organ-wide gene expression and cell atlas of the developing human heart. Cell 179, 1647–1660.e19 (2019). 31835037 10.1016/j.cell.2019.11.025

[36] I. Eralp, H. Lie-Venema, N. A. M. Bax, M. C. E. F. Wijffels, A. van der Laarse, M. C. Deruiter, A. J. J. C. Bogers, N. M. S. van den Akker, R. G. Gourdie, M. J. Schalij, R. E. Poelmann, A. C. Gittenberger-de Groot, Epicardium-derived cells are important for correct development of the Purkinje fibers in the avian heart. Anat. Rec. A Discov. Mol. Cell. Evol. Biol. 288, 1272–1280 (2006). 17075847 10.1002/ar.a.20398 PMC2610390

[37] H. Hu, S. Lin, S. Wang, X. Chen, The role of transcription factor 21 in epicardial cell differentiation and the development of coronary heart disease. Front. Cell Dev. Biol. 8, (2020). 10.3389/fcell.2020.00457 PMC7290112 32582717

[38] A. Andersson, L. Larsson, L. Stenbeck, F. Salmén, A. Ehinger, S. Z. Wu, G. al-Eryani, D. Roden, A. Swarbrick, Å. Borg, J. Frisén, C. Engblom, J. Lundeberg, Spatial deconvolution of HER2-positive breast cancer delineates tumor-associated cell type interactions. Nat. Commun. 12, 6012 (2021). 34650042 10.1038/s41467-021-26271-2 PMC8516894

[39] S. Z. Wu, G. al-Eryani, D. L. Roden, S. Junankar, K. Harvey, A. Andersson, A. Thennavan, C. Wang, J. R. Torpy, N. Bartonicek, T. Wang, L. Larsson, D. Kaczorowski, N. I. Weisenfeld, C. R. Uytingco, J. G. Chew, Z. W. Bent, C. L. Chan, V. Gnanasambandapillai, C. A. Dutertre, L. Gluch, M. N. Hui, J. Beith, A. Parker, E. Robbins, D. Segara, C. Cooper, C. Mak, B. Chan, S. Warrier, F. Ginhoux, E. Millar, J. E. Powell, S. R. Williams, X. S. Liu, S. O’Toole, E. Lim, J. Lundeberg, C. M. Perou, A. Swarbrick, A single-cell and spatially resolved atlas of human breast cancers. Nat. Genet. 53, 1334–1347 (2021). 34493872 10.1038/s41588-021-00911-1 PMC9044823

[40] M. C. Hung, A. L. Schechter, P. Y. Chevray, D. F. Stern, R. A. Weinberg, Molecular cloning of the neu gene: Absence of gross structural alteration in oncogenic alleles. Proc. Natl. Acad. Sci. U.S.A. 83, 261–264 (1986). 3001730 10.1073/pnas.83.2.261 PMC322837

[41] M. Tan, J. Yao, D. Yu, Overexpression of the c-erbB-2 gene enhanced intrinsic metastasis potential in human breast cancer cells without increasing their transformation abilities. Cancer Res. 57, 1199–1205 (1997). 9067293

[42] S. E. Moody, C. J. Sarkisian, K. T. Hahn, E. J. Gunther, S. Pickup, K. D. Dugan, N. Innocent, R. D. Cardiff, M. D. Schnall, L. A. Chodosh, Conditional activation of Neu in the mammary epithelium of transgenic mice results in reversible pulmonary metastasis. Cancer Cell 2, 451–461 (2002). 12498714 10.1016/s1535-6108(02)00212-x

[43] T. Holbro, G. Civenni, N. E. Hynes, The ErbB receptors and their role in cancer progression. Exp. Cell Res. 284, 99–110 (2003). 12648469 10.1016/s0014-4827(02)00099-x

[44] D. M. Reese, D. J. Slamon, HER-2/neuSignal transduction in human breast and ovarian cancer. Stem Cells 15, 1–8 (1997). 10.1002/stem.150001 9007217

[45] M. Tan, D. Yu, Molecular mechanisms of ErbB2-mediated breast cancer chemoresistance, in *Madame Curie Bioscience Database [Internet]* (Landes Bioscience, 2000–2013); www.ncbi.nlm.nih.gov/books/NBK6194/.

[46] G. Curigliano, D. Disalvatore, A. Esposito, G. Pruneri, M. Lazzeroni, A. Guerrieri-Gonzaga, A. Luini, R. Orecchia, A. Goldhirsch, N. Rotmensz, B. Bonanni, G. Viale, Risk of subsequent in situ and invasive breast cancer in human epidermal growth factor receptor 2-positive ductal carcinoma in situ. Ann. Oncol. 26, 682–687 (2015). 25600567 10.1093/annonc/mdv013

[47] M. A. Thorat, P. M. Levey, J. L. Jones, S. E. Pinder, N. J. Bundred, I. S. Fentiman, J. Cuzick, Prognostic and predictive value of HER2 expression in ductal carcinoma in situ: Results from the UK/ANZ DCIS randomized trial. Clin. Cancer Res. 27, 5317–5324 (2021). 34380636 10.1158/1078-0432.CCR-21-1239 PMC7612534

[48] T. Cooke, J. Reeves, A. Lanigan, P. Stanton, HER2 as a prognostic and predictive marker for breast cancer. Ann. Oncol. 12(suppl. 1), S23–S28 (2001). 11521717 10.1093/annonc/12.suppl_1.s23

[49] D. Gajria, S. Chandarlapaty, HER2-amplified breast cancer: Mechanisms of trastuzumab resistance and novel targeted therapies. Expert Rev. Anticancer Ther. 11, 263–275 (2011). 21342044 10.1586/era.10.226 PMC3092522

[50] X. Li, M. Wang, T. Gong, X. Lei, T. Hu, M. Tian, F. Ding, F. Ma, H. Chen, Z. Liu, A S100A14-CCL2/CXCL5 signaling axis drives breast cancer metastasis. Theranostics 10, 5687–5703 (2020). 32483412 10.7150/thno.42087 PMC7255008

[51] L. Fang, Y. Wang, Y. Gao, X. Chen, Overexpression of CXXC5 is a strong poor prognostic factor in ER+ breast cancer. Oncol. Lett. 16, 395–401 (2018). 29928427 10.3892/ol.2018.8647 PMC6006432

[52] V. Kleshchevnikov, A. Shmatko, E. Dann, A. Aivazidis, H. W. King, T. Li, R. Elmentaite, A. Lomakin, V. Kedlian, A. Gayoso, M. S. Jain, J. S. Park, L. Ramona, E. Tuck, A. Arutyunyan, R. Vento-Tormo, M. Gerstung, L. James, O. Stegle, O. A. Bayraktar, Cell2location maps fine-grained cell types in spatial transcriptomics. Nat. Biotechnol. 40, 661–671 (2022). 35027729 10.1038/s41587-021-01139-4

[53] S. Codeluppi, L. E. Borm, A. Zeisel, G. la Manno, J. A. van Lunteren, C. I. Svensson, S. Linnarsson, Spatial organization of the somatosensory cortex revealed by osmFISH. Nat. Methods 15, 932–935 (2018). 30377364 10.1038/s41592-018-0175-z

