# Supplementary material for: Cell composition inference and identification of layer-specific spatial transcriptional profiles with POLARIS
Source: Sci Adv. 2023 Mar 1;9(9):eadd9818. doi: 10.1126/sciadv.add9818 (PMC9977174; doi:10.1126/sciadv.add9818)
Supplement: Supplementary file 1 — Figs. S1 to S8 [file sciadv.add9818_sm.pdf]

Science Advances
AAAS

Supplementary Materials for

Cell composition inference and identification of layer-specific spatial transcriptional profiles with POLARIS

Jiawen Chen *et al.*

Corresponding author: Yun Li, yunli@med.unc.edu

Sci. Adv. **9**, eadd9818 (2023)
DOI: 10.1126/sciadv.add9818

This PDF file includes:

Figs. S1 to S8

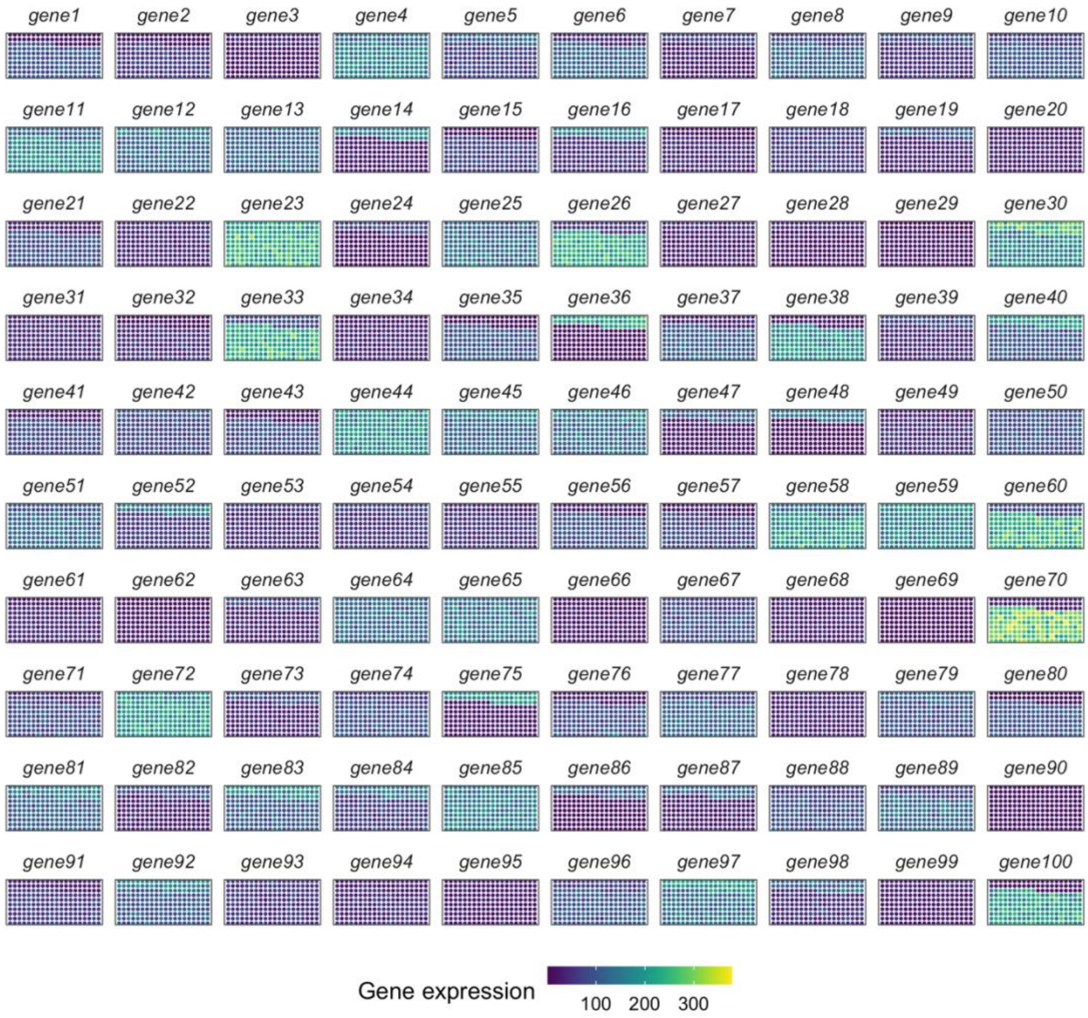

Fig. S1 | Simulated gene expression pattern across layers. The points are colored by gene expression levels. The layer structure is same as **Fig. 2a**.

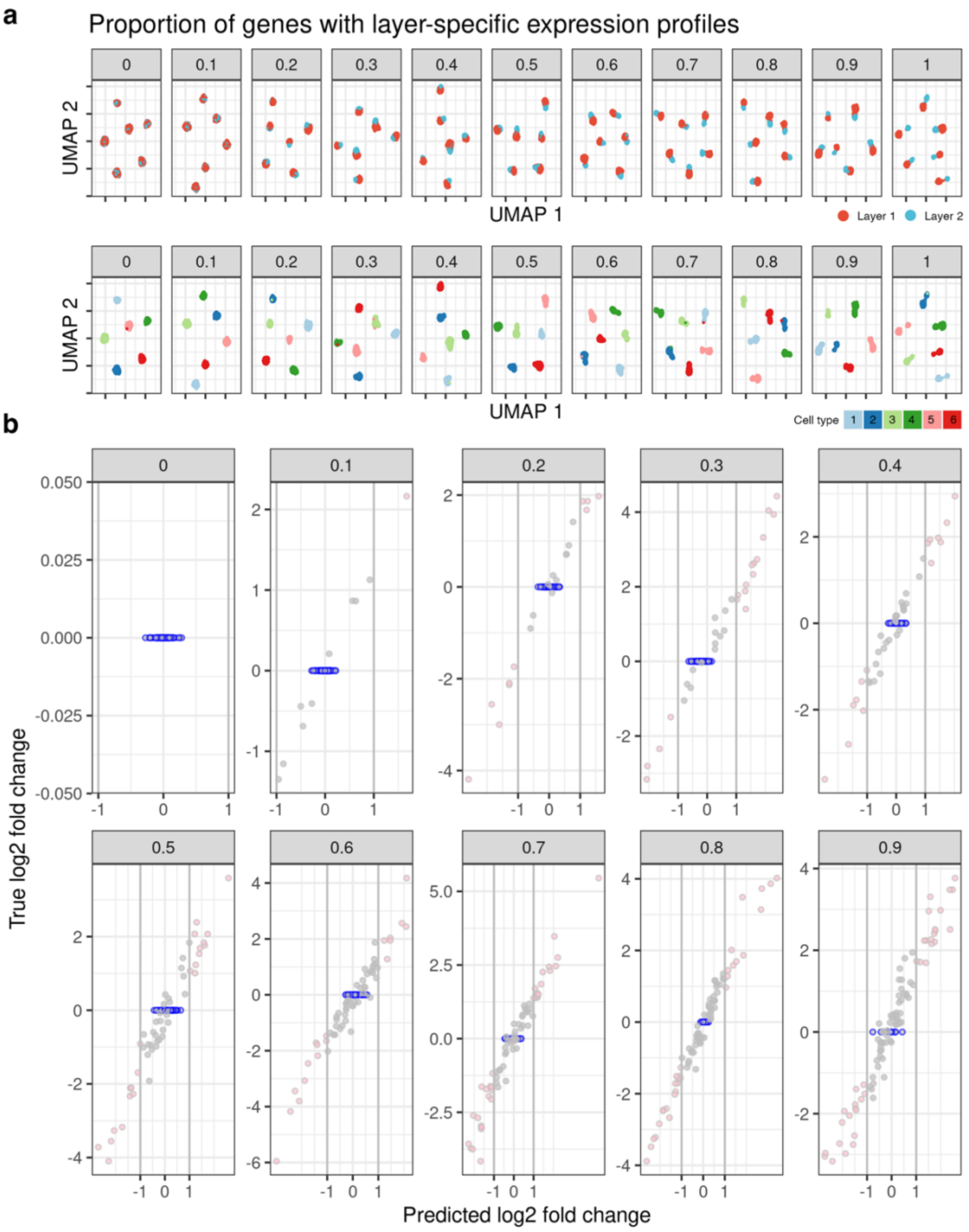

Fig. S2 | POLARIS inferred log2 fold change of gene expression across layers agrees with the true log2 fold change and well-controlled type-I error in simulations. (a) UMAP visualization of cells colored by layer (top) and cell type (bottom) (b) Points with blue borders are the genes with invariant gene expression profiles across layers. Pink points mark LDE genes identified by POLARIS.

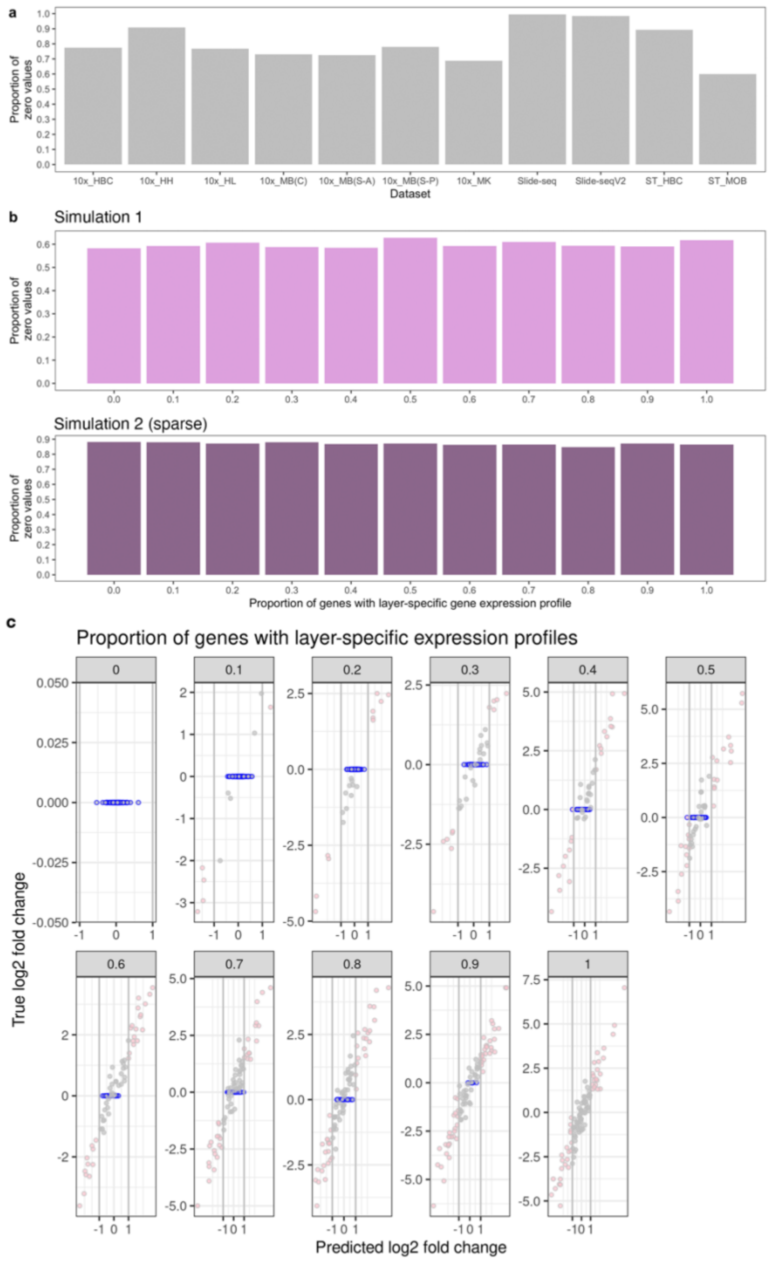

Fig. S3 | Sparse simulation results. (a) The sparsity of real ST datasets. The numbers are from Zhao et al. [56]. (b) (top) the sparsity in simulation 1 (bottom) the sparsity in simulation 2. (c) POLARIS inferred log2 fold change of gene expression across layers agrees with the true log2 fold change and well-controlled type-I error in sparse simulations.

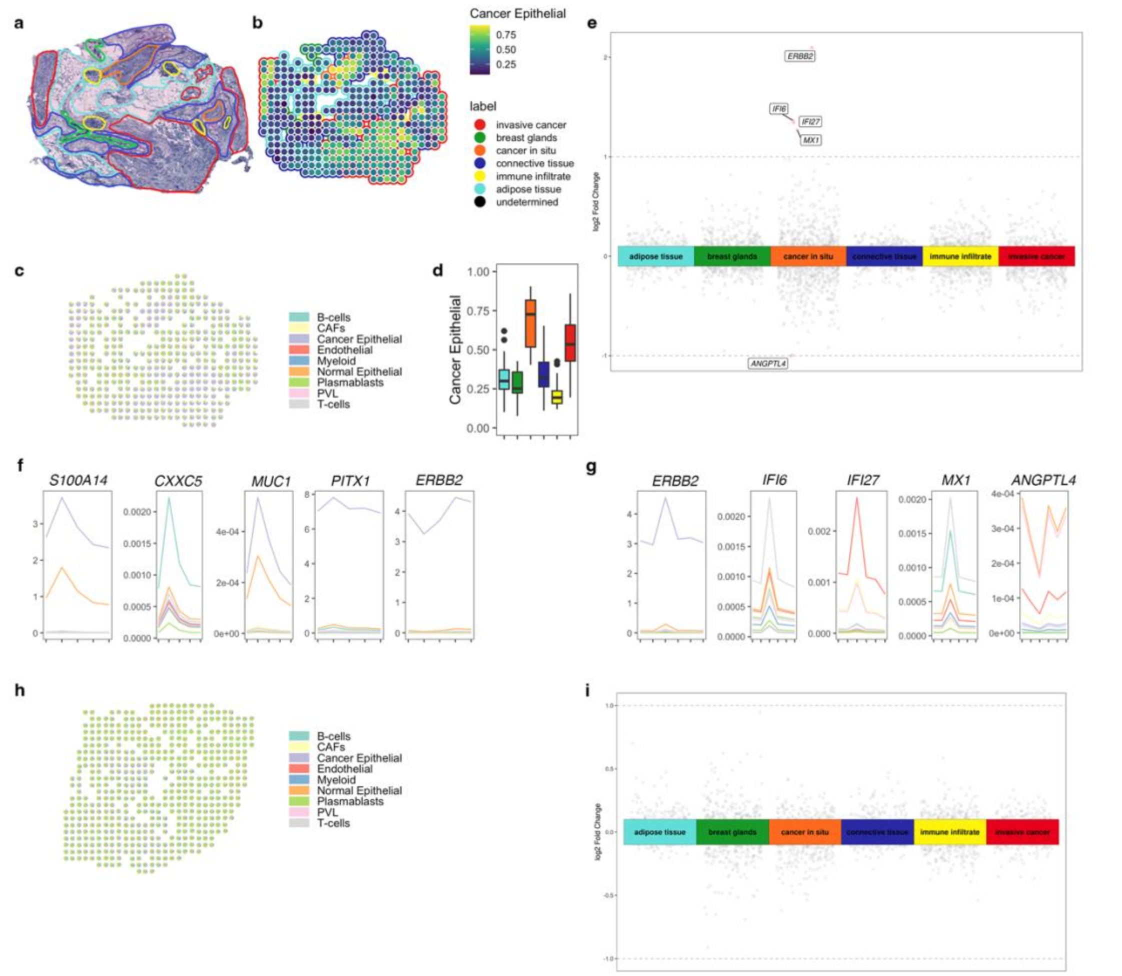

Fig. S4 | POLARIS inference on breast cancer. On slide G2: (a) Pathologist annotation on slide G2 (b) POLARIS inferred cancer epithelial cell proportion (c) POLARIS inferred cell composition (d) Distribution of POLARIS inferred cancer epithelial cell proportions, in each layer (color scheme is the same as in a and b above) (e) POLARIS inferred log2 fold change of gene expression across layers. Points with absolute value greater than 1 are colored as pink, otherwise, gray. (f) POLARIS inferred gene expression location parameter on slide A1 (Fig. 3f-k) (g) POLARIS inferred gene expression location parameter on this slide G2. **On slide H1:** (h) POLARIS inferred cell composition (i) POLARIS inferred log2 fold change of gene expression across layers.

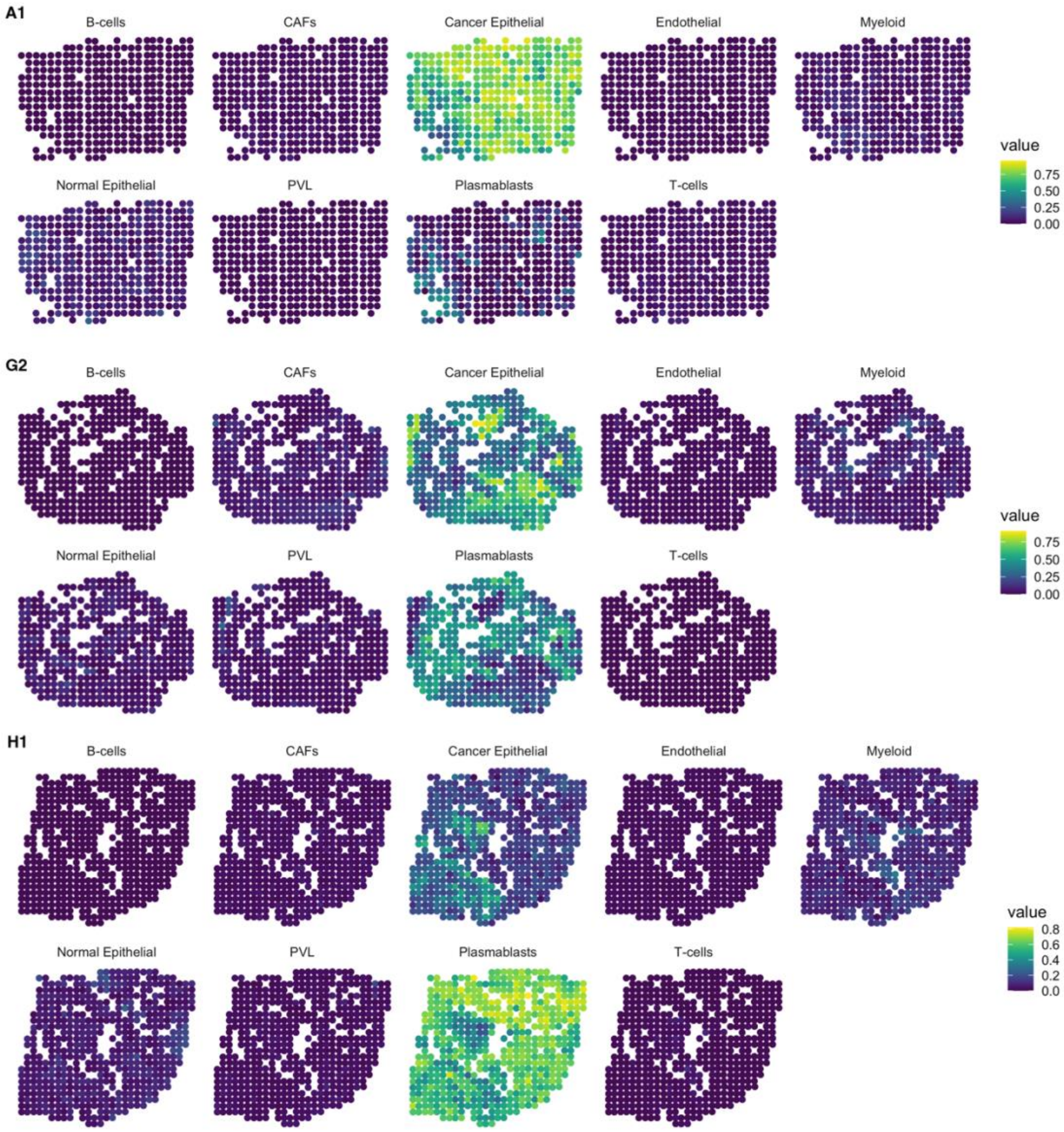

Fig. S5 | POLARIS inferred cell composition on breast cancer patients. (top) on slide A1 (middle) on slide G2 (bottom) on slide H1.

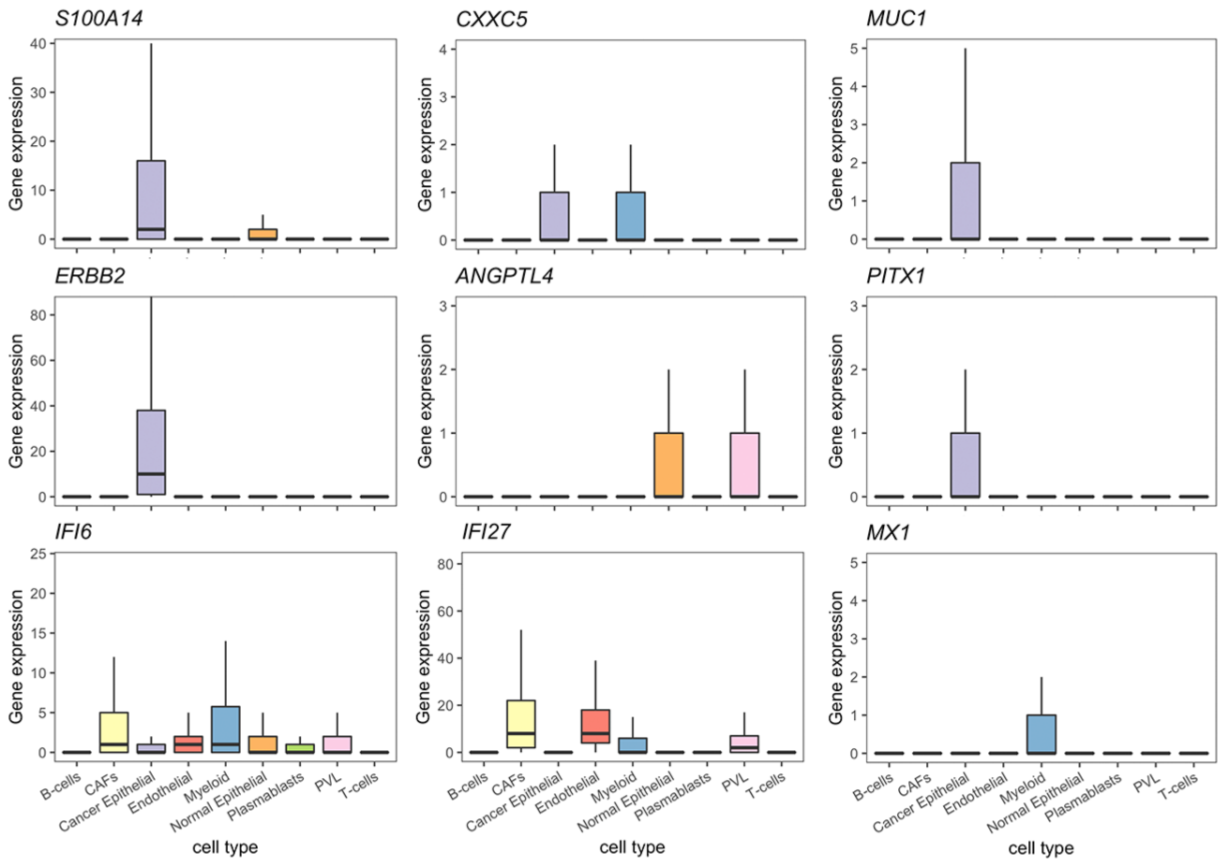

Fig. S6 | Cell type specific gene expression distribution of POLARIS identified LDE genes in breast cancer, based on scRNA-seq data. The side-by-side boxes are colored by cell types.

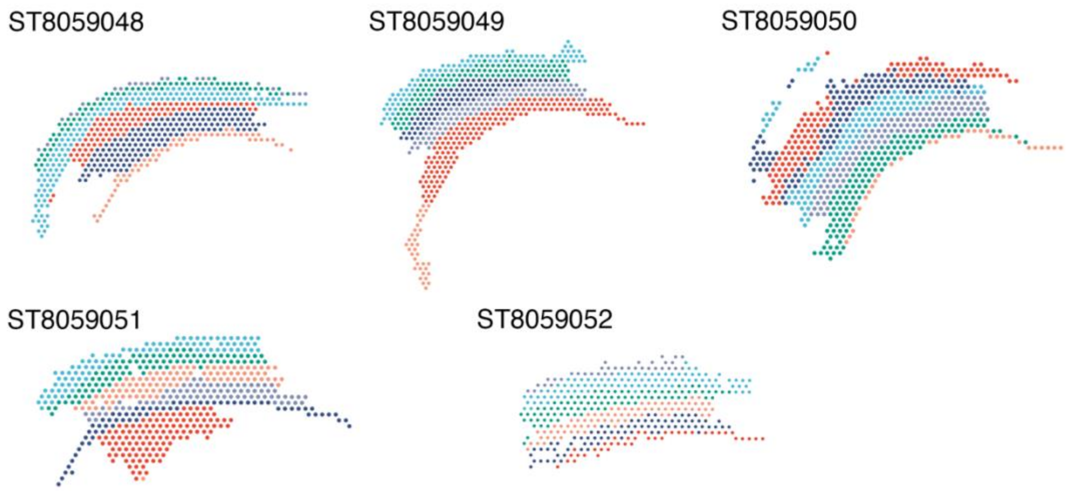

Fig. S7 | BayesSpace inferred clusters in the mouse cortex SSp 10x Visium data.

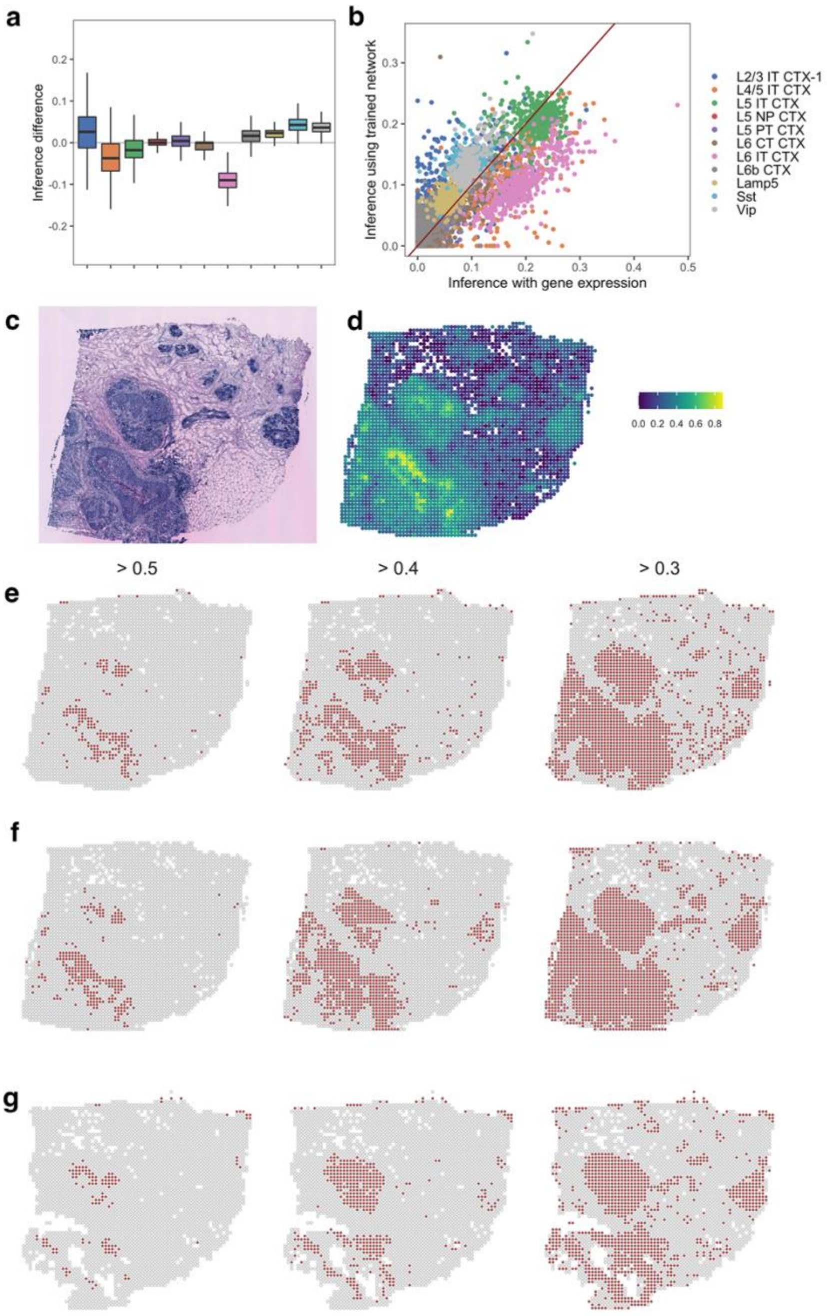

Fig. S8 | Cell composition inferred using POLARIS-trained image network is comparable with inference with gene expression and can be used as a new method for tissue segmentation. (a) Difference in the inferred cell composition, between estimates using POLARIS-trained image network and estimates using gene expression, former minus latter. Each boxplot represents one cell type. (b) Estimated cell type proportions from POLARIS-trained image network and from gene expression data are correlated (for example, Pearson's correlation in L2/3 IT CTX-1 : 0.52, L4/5 IT CTX : 0.75, L5 IT CTX : 0.21, L5 NP CTX : 0.29, L5 PT CTX : 0.32, L6 CT CTX : 0.76, L6 IT CTX : 0.78, L6b CTX : 0.80, Lamp5 : 0.36, Sst : 0.31, Vip : 0.69). (c) Histology image of HER2+ breast cancer slide H2 (d) Super-resolution inference of H2 using POLARIS image network trained on H1. (e-g) Tissue registration using different cancer epithelial cell

proportion (>0.5, >0.4, >0.3) on slide (e) H1 (f) H2 (g) H3. Spots colored in red have a larger proportion of cancer epithelial cells than the corresponding threshold (0.5, 0.4, and 0.3 from left to right). These spots can be treated as falling in cancerous areas.
